# Nucleosome Positioning and NDR Structure at RNA Polymerase III Promoters

**DOI:** 10.1038/srep41947

**Published:** 2017-02-08

**Authors:** Alexandra Søgaard Helbo, Fides D. Lay, Peter A. Jones, Gangning Liang, Kirsten Grønbæk

**Affiliations:** 1 Department of Hematology, Rigshospitalet, Faculty of Health Sciences, University of Copenhagen, Copenhagen, 2100, Denmark; 2 Department of Urology, Norris Comprehensive Cancer Center, Keck School of Medicine, University of Southern California, Los Angeles, 90089, USA; 3 Van Andel Research Institute, Grand Rapids, 49503, USA

## Abstract

Chromatin is structurally involved in the transcriptional regulation of all genes. While the nucleosome positioning at RNA polymerase II (pol II) promoters has been extensively studied, less is known about the chromatin structure at pol III promoters in human cells. We use a high-resolution analysis to show substantial differences in chromatin structure of pol II and pol III promoters, and between subtypes of pol III genes. Notably, the nucleosome depleted region at the transcription start site of pol III genes extends past the termination sequences, resulting in nucleosome free gene bodies. The +1 nucleosome is located further downstream than at pol II genes and furthermore displays weak positioning. The variable position of the +1 location is seen not only within individual cell populations and between cell types, but also between different pol III promoter subtypes, suggesting that the +1 nucleosome may be involved in the transcriptional regulation of pol III genes. We find that expression and DNA methylation patterns correlate with distinct accessibility patterns, where DNA methylation associates with the silencing and inaccessibility at promoters. Taken together, this study provides the first high-resolution map of nucleosome positioning and occupancy at human pol III promoters at specific loci and genome wide.

Transcription in eukaryotes is carried out by multiple DNA-dependent RNA polymerases, pol I, pol II and pol III. Pol I transcribes ribosomal RNAs, pol II transcribes mainly protein-encoding genes, while pol III transcribes genes encoding short (typically <200 bp), non-coding RNAs^1^. Transcription by pol III is closely associated with growth and proliferation as the gene products are directly involved in processes such as translation and splicing. Extensive work on pol III genes has revealed the factors required for directing pol III to target genes^1^,^2^,^3^, and has defined the three types of pol III genes based on (a) presence and position of cis-regulatory promoter elements and (b) the requirement for specific transcription factors (TFs) in the pol III pre-initiation complex. All pol III transcription requires the TFIIIB complex, which recognizes the promoter through the TATA binding protein and then recruits pol III. In brief, 5S rRNA is the sole Type I gene, and utilizes TFIIIA and TFIIIC, which recognizes the internal A and C boxes. tRNAs are the major gene class in Type II genes, and require TFIIIC to bind the internal A and B box promoter elements to initiate transcription. Type III genes (e.g. U6) resemble pol II gene promoters by having external promoter elements, which are composed of distal and proximal sequence elements (DSE and PSE) as well as a TATA box.

The nucleosome is the fundamental unit of chromatin, consisting of 146 bp DNA wrapped around a histone core^4^, and is directly involved in gene regulation by determining access of TFs to the underlying promoter elements and by stabilizing protein interactions. Occupancy by nucleosomes in functional regions such as gene promoters or enhancers, is correlated with gene silencing^5^. Furthermore, global studies have provided much insight into the nucleosome positioning around promoters of expressed pol II genes, which generally exhibit a nucleosome depleted region (NDR) at the transcription start site (TSS), and a strongly positioned +1 nucleosome with downstream nucleosome phasing^6^,^7^,^8^,^9^,^10^. The main determinant of nucleosome positioning is believed to be trans-acting remodelers, but the underlying DNA sequence may favor certain positions (reviewed in refs 11 and 12).

Early studies showing that pol III genes were constitutively expressed and devoid of nucleosomes contributed to the notion that chromatin did not regulate pol III genes^9^,^13^. However, multiple studies, many of which were performed in yeast, have since identified regulatory roles of chromatin and nucleosome occupancy in pol III transcription^8^,^14^,^15^,^16^,^17^,^18^,^19^,^20^,^21^. While the pol III transcriptional machinery has intrinsic histone acetyltransferase activity^22^, studies have shown that chromatin remodelers are actively associated with pol III transcription^17^,^23^,^24^,^25^. In addition, both active and inactive pol III gene promoters exhibit similar histone mark profiles to that of active and inactive pol II gene promoters, respectively^20^,^26^,^27^,^28^, which however are deposited more strongly upstream of the TSS, compared to downstream, at pol III genes^20^.

Here, we generated the first high resolution map of nucleosome positioning at pol III promoters in human cells to examine the differences and similarities between pol II and III promoters, as well as within pol III promoter subtypes.

## Results

### Genome wide analysis of nucleosome positioning at pol III promoters

**Figure 1:**
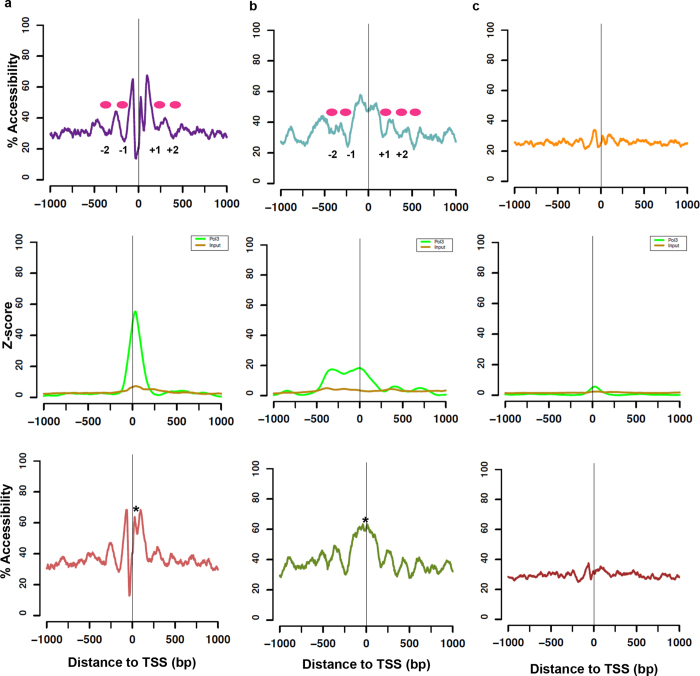
Genome wide NOMe-seq reveals differential positioning patterns of nucleosomes at active pol III promoters. NOMe-seq was performed on K562 cells and % accessibility was averaged across active promoters of (**a**) active tRNA genes (**b**) active non-tRNA genes, and (**c**) inactive tRNA genes. The position of the nucleosomes are indicated between the accessibility peaks (pink ovals), which are between 150–200 bp. Top panels indicate accessibility plots across the promoters. Middle panels indicate the pol III z-score enrichment across the promoters. Bottom panels represent NOMe-seq on K562 cells treated with 400 mM NaCl. Regions that change in accessibility following NaCl treatment are indicated by * and are associated with increased TSS accessibility.

We employed the NOMe-seq assay developed by our group to generate a genome wide map of chromatin accessibility in K562 cells to study the chromatin landscape of active promoters. Freshly isolated nuclei were treated with M.CviPI, an enzyme that methylates cytosines within GpC dinucleotides that are not protected by nucleosomes or tightly bound proteins^29^. Chromatin accessibility is determined by calculating the percentage of GpC methylation across all reads covering the position, which in turn infers nucleosome occupancy/positioning patterns. Regions of inaccessibility less than 146 bp in size are termed “footprints”. We first examined the nucleosome positioning at pol III gene promoters (Fig. 1). As the majority of active pol III genes in K562 cells are tRNAs (203/225, Table S1), we further subdivided active pol III promoters into tRNA (Fig. 1a), non-tRNA groups (Fig. 1b) and inactive tRNAs (Fig. 1c).

Active tRNA gene promoters, which are Type II genes that contain internal promoter elements, exhibit an NDR with a well-positioned −1 nucleosome (centered at −150 bp) and a +1 nucleosome (centered at +220 bp), as well as upstream/downstream nucleosome phasing. This phasing becomes less apparent past the −2/+2 nucleosomes (Fig. 1a, top panel). This positioning is different from that at active pol II genes, where the prominent feature is an NDR delineated by a very highly positioned +1 nucleosome centered at +125 bp (Fig. S1a). This positioning pattern around the NDR at tRNA genes is consistent with findings in yeast^30^,^31^, and we now expand this to also encompass human cells. At tRNA promoters, we find two distinct regions of inaccessibility within the NDR; a prominent peak immediately upstream of the TSS and a slightly less pronounced peak immediately downstream of the TSS. Both of these footprints do not represent nucleosomes due to their smaller size, but could represent the binding of TFs. Interestingly, we found that these NDR-associated footprints mapped to the genomic location of pol III enrichment at these loci (Fig. 1a, middle panel). To further investigate whether these footprints are the result of potential TF or pol III binding, we performed NOMe-seq on K562 nuclei treated with 400 mM NaCl, which removes chromatin associated proteins, but not histones from the chromatin, hence maintaining nucleosome positioning and phasing patterns^32^ (Fig. S1b). Upon NaCl treatment, we observed a gain in accessibility at the downstream footprint, while the upstream footprint was unaffected (Fig. 1a lower panel, Fig. S2a and d). The downstream footprint is most likely the result of pol III binding, which is most enriched here (Fig. 1a middle panel), and potentially also due to TFIIIC binding. Previous reports in yeast have shown that TFIIIB binding occurs immediately upstream of the TSS and is highly resistant to NaCl wash^33^. Therefore, the upstream NaCl-resistant peak likely represents the footprint of TFIIIB.

The NDR structure and nucleosome positioning patterns at non-tRNAs are distinct from that in the tRNA group (Fig. 1b), where the majority of non-tRNA genes have external promoter elements (as they are mainly Type III genes, but also Type I). In the non-tRNA group we again observed a footprint within the NDR at the TSS, albeit less pronounced than at tRNA genes. Moreover, the −1 nucleosome is located further upstream (centered at −250 bp) while the +1 nucleosome is centered at +125 bp (similar to pol II, Fig. S1a). Pol III enrichment is more broadly distributed across the TSS (Fig. 1b, middle panel), which may explain the difference in TSS-footprints between tRNA and non-tRNA groups. NaCl treatment of the nuclei resulted in loss of the TSS-associated binding proteins (Fig. 1b lower panel, Fig. S2b,c), again suggesting that pol III binding across the TSS contributes to the footprint.

Unlike yeast, where all tRNA promoters are occupied and expressed^34^, human tRNAs are expressed in a cell type- and process specific manner (e.g. proliferating vs. differentiating)^26^,^27^,^35^. We analyzed the chromatin landscape at inactive tRNAs in K562 (397 tRNAs, Fig. 1c). Promoters of inactive genes show low accessibility, which is not affected by high concentrations of NaCl, and a lack of pol III enrichment across the promoters (Fig. 1c top vs. lower panel).

Our data shows that the coding regions of pol III genes are devoid of nucleosomes, which is in accordance with previous studies^20^,^27^. Consequently, in contrast to pol II genes that exhibit nucleosomes within the gene body, the +1 nucleosome of pol III genes appears to be located at the 3′ end of transcripts, or downstream of the pol III transcription termination sites (TTSs). Furthermore, we hypothesized that the weak positioning of the +1 nucleosome observed in the genome wide plots might result from differences in pol III transcript length. To gain insight into the possible biological role of the +1 nucleosome at pol III genes, we analyzed the nucleosome positioning patterns relative to the TTS of all active pol III genes (Fig. S1c). The TTS lies within the NDR, immediately after the second NDR-associated footprint. The +1 nucleosome is centered at +125 bp relative to the TTS. Aligning the NOMe-seq data to the TTS does not reveal an increase in +1 nucleosome positioning, nor a decrease in the strength of the positioning of the −1 nucleosome, which is similar to data found at yeast tRNAs^30^. Hence, active pol III genes are nucleosome depleted within the gene body irrespective of transcript length, indicating that the biological role of the +1 nucleosome may be different from pol II genes, i.e. the +1 nucleosome regulates transcriptional activation/suppression at pol III genes^30^,^36^, while it controls promoter-proximal pausing at pol II genes^37^,^38^,^39^.

Moreover, in general, differences in gene size is a prominent characteristic between pol II and III genes, where the majority of pol III genes are only a few hundred bases long, compared to several thousands for a typical pol II gene. Hence, transcript size could be an important determinant of the nucleosome positioning, and potentially explain some of the positioning differences we observe between active pol II and III genes. To examine this further, we analyzed accessibility specifically at pol II genes <100 bp in length, in order to mimic the size of pol III genes (Fig. S1d,e). Here, it is seen that the set of short pol II genes (n = 69) exhibit a distinct positioning profile compared to a randomized set (n = 69) of ‘regular’ length pol II genes. The short pol II gene profile is reminiscent of that of the non-tRNA promoters (Fig. 1b). This could indicate that the length of the gene is important for the positioning characteristics, and that the position of the +1 nucleosome may also be dependent on gene length. Therefore, while descriptive, this data indicates that it may not be the specific polymerase, or the accessory proteins, which solely regulate positioning, but rather gene length and promoter element composition together.

Taken together, the data suggests that the distinctive features between active pol II and pol III promoters are 1) the location and occupancy of the +1 nucleosome and 2) depletion of nucleosomes of active pol III gene promoters and bodies. While further validation is required, this data indicates that similar mechanisms govern positioning at pol II and III genes, and that promoter element usage and gene length also are important determinants of nucleosome positioning. In addition, the chromatin structures at the tRNA and non-tRNA groups differ markedly, also indicating that the promoter element composition may affect positioning patterns.

### Detailed nucleosome mapping at pol III promoter subtypes by high resolution mapping of specific loci

To verify the genome wide observations, and further examine the differences in nucleosome positioning in pol III promoter subtypes, we extended the study to encompass three additional human cell lines (malignant and non-malignant) and performed a modified, high resolution NOMe-seq analysis at specific loci representing Type I/II (internal) promoters and Type III (external) pol III promoters. The modified NOMe-seq analysis combines M.CviPI and M.SssI enzymes, the latter of which methylates cytosines in CpG dinucleotides that are not endogenously methylated^40^. By analyzing accessibility information of both GpC and CpG sites, we can increase the resolution, which is advantageous at loci where the GpC distribution is lower. However, this method can only be used on regions where endogenous CpG sites are unmethylated (Figs S3 and 4). Analysis of chromatin accessibility at specific loci complements our genome wide analysis, allowing for the study of nucleosome positioning and occupancy at the level of individual DNA molecules.

**Figure 2:**
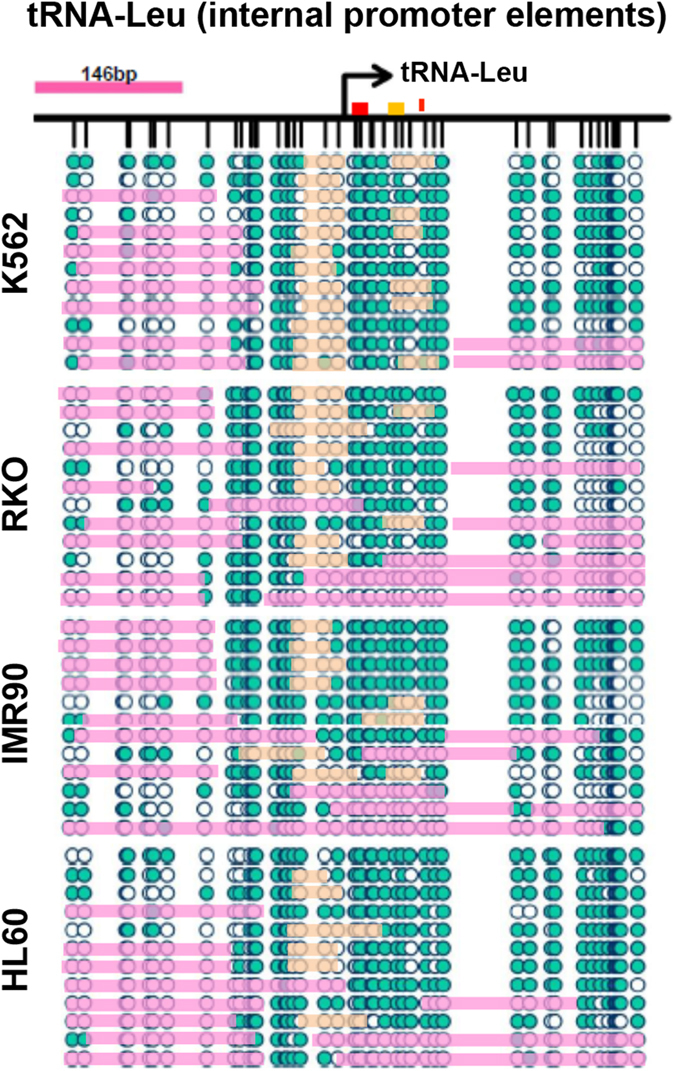
High resolution accessibility mapping of the promoter structure at tRNA-Leu. Loci-specific analysis was performed at tRNA-Leu (chromosome 14) (Type II internal promoter). To increase resolution, a modified NOMe-seq treatment that employs a combination of M.CviPI and M.SssI was performed on nuclei from four cell lines (K562, RKO, IMR90 and HL60). The resulting combinatory pattern of methylation states generates a map of chromatin accessibility and nucleosome positioning as well as occupancy at individual DNA molecules. Teal circles represent accessible GpC/CpC sites, white inaccessible. If regions of consecutive inaccessible sites equal or exceed 146 bp, we indicate the region as being occupied by a nucleosome (pink bars). Orange bars indicate regions of inaccessibility shorter than 146 bp, i.e. footprints at the TSS. Promoter elements, A and B boxes, are indicated by red and orange boxes, the red line indicates the TTS.

We first examined the nucleosome positioning at tRNA-Leu, a canonical Type II gene with internal promoter elements. We observed an NDR ranging from −100 to >+200 bp that extends across the internal promoter elements in all cell types (Fig. 2). The distal edge of the −1 nucleosome is found at −100 bp, and exhibits high occupancy across cell types (occupancy at 8/12 molecules analyzed in all cell types). In contrast, the +1 nucleosome shows lower occupancy (occupancy of 3–6/12 molecules per cell type). The distal edge of the +1 nucleosome is located at +200 bp or further downstream (beyond the PCR fragment analyzed), which is distinct from pol II promoters^29^, and may allow access to the A and B box promoter elements. Interestingly, in yeast, the RSC chromatin remodeler allows access to the internal promoter elements and termination sequences through active remodeling of the +1 nucleosome^17^,^23^,^30^, and our data suggests that human tRNAs may be regulated by a similar mechanism, while nucleosome occupancy of the internal promoter elements and/or TTS are most likely silenced^30^,^41^.

**Figure 3:**
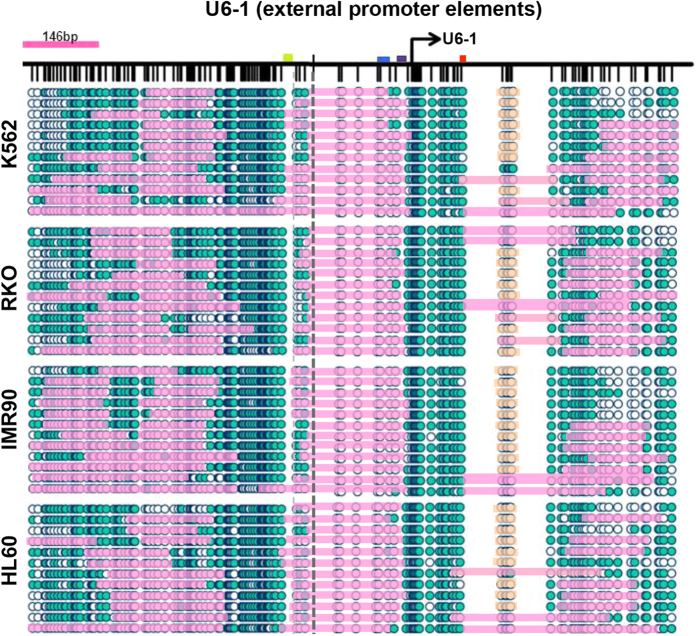
High resolution accessibility mapping of the promoter structure at U6. High resolution NOMe-seq map at U6-1 (Type III gene, external promoter) in K562, RKO, IMR90 and HL60 cells. Teal circles represent accessible GpC/CpC sites, white inaccessible. If regions of consecutive inaccessible sites equal or exceed 146 bp, we indicate the region as being occupied by a nucleosome (pink bars). Orange bars indicate regions of inaccessibility shorter than 146 bp, i.e. footprints, here at the TTS. The TATA box is indicated by a purple box, PSE by blue and DSE by green. Red line indicates TTS. Dotted line indicates the junction between the two separate PCR fragments analyzed.

We next examined the structure of the non-tRNA promoter of RNU6-1, which is a Type III gene containing external promoter elements (Fig. 3). Consistent with the genome wide data, the nucleosome positioning at this Type III gene promoter is different to that of Type II genes. Firstly, it is apparent that the −1 nucleosome exhibits high occupancy (12/12 molecules in all cell types) as well as positioning immediately upstream of the TSS (−20 to −170 bp), in the genomic region between the DSE and PSE promoter elements, in all cell types and all DNA molecules analyzed. This is in agreement with a prior report showing that the −1 nucleosome facilitates looping of the DSE to PSE element to initiate transcription^16^. While the authors of that publication were unable to define the precise location of the −1 nucleosome borders, we now report the exact position of this nucleosome in human cells. Secondly, the upstream −2 and −3 nucleosomes exhibit weak positioning, both within and between cell types. A ~150 bp region of open chromatin is observed between the −1 and −2 nucleosomes, and may allow TF binding to the DSE, while the region between the −2 and −3 nucleosomes varies between 0–150 bp. An NDR (−20 bp to approx. +300 bp) is observed in all cell types, with the +1 nucleosome proximal edge located at approximately +300 bp, showing high occupancy (7–9/12 molecules occupied), but never at the TSS (as was seen at tRNA-Leu). In contrast to tRNA-Leu, there is no TSS-associated footprint, which may reflect the differential promoter element usage. However, a footprint downstream of the TTS (+150 bp, indicated in orange) is observed. Due to low GpC/CpG coverage in this region, we cannot be sure that this footprint represents a nucleosome, which would correlate with the location of the +1 nucleosome at non-tRNA genes from Fig. 1e.

Taken together, these data extend our genome wide observations and provide the first detailed map of nucleosome occupancy and positioning across multiple human cell lines at specific pol III promoter subtypes at the level of individual DNA molecules. Our data confirms that the nucleosome positioning and occupancy patterns appear highly dependent on the location of the underlying promoter sequences (i.e. internal vs. external promoter elements) that may orchestrate the nucleosomal arrangements as well as the binding of the transcriptional machinery.

### Nucleosome mapping relative to gene expression and DNA methylation

We next examined if the positioning of nucleosomes around the pol III promoter could be correlated with expression patterns. Due to the repetitive nature and high sequence similarities of many pol III genes, expression analysis at individual genes is challenging. We therefore examined the expression and promoter accessibility around vtRNA1-1, vtRNA1-3 and nc886 (previously annotated as vtRNA2-1) genes, where we previously have documented unique expression primers^42^,^43^. The vtRNA family of ncRNAs require both internal and external promoter elements for their transcription^44^. Previous studies have reported that this unique combination of promoter elements may account for the tissue specific expression patterns observed at these transcripts, which share a high degree of sequence similarity^44^,^45^,^46^. However, as the vtRNAs contain the same promoter elements, additional cis- and trans-regulatory factors are most likely involved in the regulation of individual vtRNA genes.

**Figure 4:**
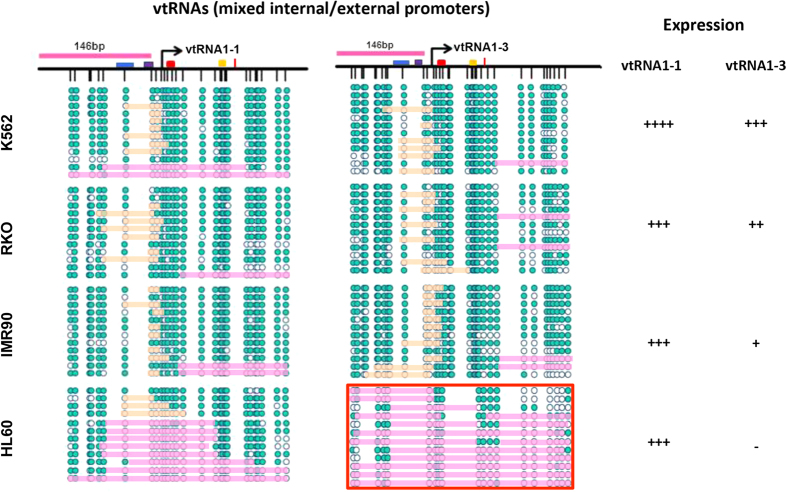
High resolution accessibility mapping of the promoter structure at vtRNA1-1 and vtRNA1-3. Left panels: High resolution NOMe-seq map at vtRNA1-1 and vtRNA1-3 (mixed internal/external promoters) in K562, RKO, IMR90 and HL60 cells. In HL60, vtRNA1-3 is silenced by endogenous DNA methylation^42^ and the plot thus only represents GpC sites. Teal circles represent accessible GpC/CpC sites, white inaccessible (only GpC sites for vtRNA1-3 in HL60). If regions of consecutive inaccessible sites equal or exceed 146 bp, we indicate the region as being occupied by a nucleosome (pink bars). Orange bars indicate regions of inaccessibility shorter than 146 bp, i.e. footprints at the TSS. Blue box indicates the PSE, purple box the TATA box, red box the A box, yellow the B box and the red line indicates the TTS. Right panels: indicate relative expression levels of each vtRNA in each cell line.

We observe differential expression of vtRNA1-1 and vtRNA1-3 in the four human cell lines (Fig. 4 right panel and Fig. S5a,b). Across all cell lines, vtRNA1-1 is more highly expressed compared to vtRNA1-3 and both vtRNAs are most highly expressed in K562 cells. As previously reported, vtRNA1-3 is silenced in HL60 cells by DNA methylation (Figs S4 and S5b)^42^.

We next examined chromatin accessibility patterns at the vtRNA promoters (Fig. 4 left panel). We find that the promoters are largely nucleosome depleted at expressed vtRNAs across all examined cell types. The fragments analyzed at these loci are shorter, and therefore, do not allow for the precise mapping of the –1 and +1 nucleosomes, but our data clearly highlight that the +1 nucleosome is located further downstream past the termination sites (nucleosome proximal edge at or beyond +150 bp). There are both cell type- and transcript specific differences in the position of the +1 nucleosome, similar to what was observed at the tRNA. Moreover, vtRNA1-3, which is expectedly silenced in HL60, exhibits a nucleosome occupied promoter. As this locus is endogenously methylated, we can only determine accessibility from GpC sites, which reduces the resolution at this site. Of note, the size of the footprints (orange) (Fig. S2) varies within and between cell types (between 50–75 bp), which could represent cell type and/or cell cycle specific differences in TF binding footprints, and/or the location of the transcriptional machinery. However, we do not find any noticeable correlation between promoter accessibility patterns and the vtRNA expression levels. Thus, the nucleosome occupancy and positioning patterns at cis-acting regulatory elements outside the regions analyzed may regulate the cell type specific expression. For example, when examining the upstream region of vtRNA1-1 in K562 and RKO (which exhibit a threefold difference in expression, Fig. S5a), we find that the region at the DSE is more accessible in K562 than in RKO (Fig. S6), indicating that this region is important in the transcriptional regulation and is correlated with nucleosome occupancy. Therefore, the chromatin may provide a poised state for transcription, but the expression levels and transcriptional fine-tuning may be dependent on additional TFs.

**Figure 5:**
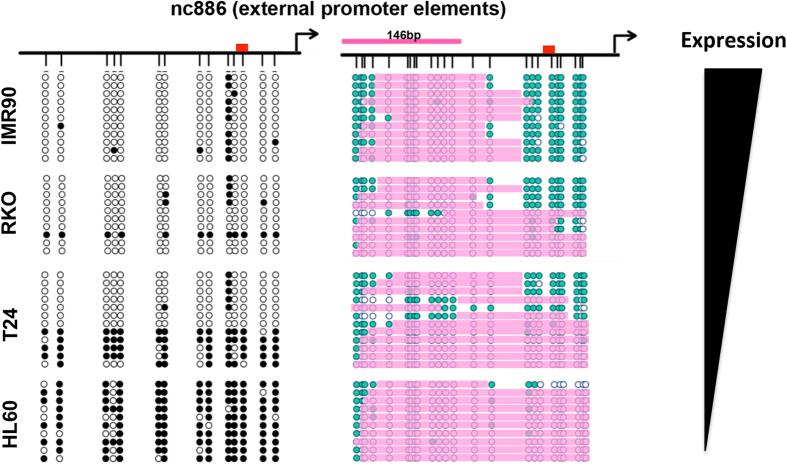
Accessibility mapping of the promoter structure at nc886. NOMe-seq at the nc886 promoter in RKO, IMR90, HL60 and T24 cells. Left panel indicates endogenous DNA methylation, right panel chromatin accessibility. Each horizontal row in each panel represents the same DNA molecule, and it is thus possible to track accessibility and DNA methylation patterns in the same DNA molecule. For the accessibility plots, teal circles represent accessible GpC sites, white inaccessible. If regions of consecutive inaccessible sites equal or exceed 146 bp, we indicate the region as being occupied by a nucleosome (pink bars). For the DNA methylation plots, white indicates unmethylated, black methylated. Red box represents the CRE-element. Relative expression of nc886 between the four cell lines is indicated in the outer right panel.

We cannot examine expression at the level of individual DNA molecules using the NOMe-seq method, but must infer patterns as exemplified above. However, if a locus contains endogenous promoter DNA methylation, it is empirically known that the region is silenced. nc886 is a pol III transcript which exhibits monoallelic methylation patterns^43^,^47^. We therefore assayed expression, chromatin accessibility and DNA methylation status at the nc886 promoter (Fig. 5, Fig. S5c). Although highly similar to the vtRNAs, nc886 lacks the TATA box, DSE or PSE elements characteristic of vtRNA promoters, but contains a cAMP-response (CRE) element in the promoter^48^,^49^. The nc886 promoter is fully methylated in HL60, monoallelically methylated (50/50) in T24 and unmethylated in RKO and IMR90 (Fig. 5 left panel). As we previously reported, nc886 is not expressed in HL60^43^ and the promoter is nucleosome occupied. Interestingly, in T24 cells, we find that methylated alleles are completely nucleosome occupied, while the unmethylated alleles show high occupancy (12/12 molecules) and positioning of the −1 nucleosome from −150 bp to −300 bp, and an NDR from −150 bp to the TSS, corresponding to the position of the CRE element. Such alleles are likely either poised for transcription or actively expressed, suggesting that the CRE element may be an important transcriptional regulator. Furthermore, while RKO and IMR90 cells both are umethylated, IMR90 cells exhibit a 2-fold higher expression (Fig. 5, right panel and Fig. S5c). Interestingly, this expression pattern correlates with the nucleosome occupancy within each cell type; half of molecules examined in RKO cells are occupied by nucleosomes, while the remaining molecules exhibit the well-positioned −1 nucleosome and an NDR at the CRE element, while all molecules exhibit the latter phenotype in IMR90 cells.

Collectively, our data at vtRNA and nc886 genes indicate that expression and methylation patterns at pol III genes correlate with nucleosome occupancy profiles around regulatory elements. While anecdotal, this suggests that the chromatin environment actively regulates pol III gene expression and future experiments will aim to uncover these interactions.

## Discussion

Pol III transcripts play a central role in governing translational output and cellular function. Therefore, gaining a basic understanding of the chromatin structure at pol III genes is important in addressing the differences in nucleosome positioning, occupancy and NDR structures of pol III promoter subtypes. Previous reports, which investigated global histone modification profiles suggested that active pol III genes were nucleosome depleted^20^,^27^. Our genome wide and loci specific NOMe-seq assays revealed that pol III genes have distinct nucleosome positioning and NDR profiles relative to pol II genes, as well as between pol III promoter classes, which are conserved across cell types (malignant vs. non-malignant and between diverse tissues of origin).

In contrast to pol II genes that have a highly positioned +1 nucleosome centered at +125 bp, the +1 nucleosome at active pol III genes displays a different structure, and is generally located further downstream of the TSS. We provide preliminary data which indicates that short pol II genes adopt similar positioning patterns to pol III, indicating that gene length may be in important regulator of positioning, irrespective of polymerase, and that the mechanistic properties of nucleosome positioning may be shared. This is in line with the recent observations that highlight a great degree of transcriptional sharing (TFs, chromatin remodelers, histone modifications, transcriptional machinery (e.g. TBP, SNAP_c_, TFIIS))^20^,^24^,^26^,^27^,^50^,^51^,^52^,^53^,^54^ between the two polymerases, emphasizing that pol II and III transcriptional systems are not unique as previously anticipated. Future studies should aim to uncover any underlying similarities in the regulation of pol II and III nucleosome positioning patterns.

The location of the +1 nucleosome appears to be highly specific to individual pol III promoter types (Fig. 1), as highlighted in detail by the differential positioning patterns between tRNA-Leu, U6 and vtRNAs. In the case of tRNA-Leu and the vtRNAs, the depletion of nucleosomes within and beyond the gene body would likely ensure access to the internal A and B box promoter elements as well as the terminal sequence. The high transcription rates of pol III genes are linked to extremely efficient initiation-elongation- termination-and-re-initiation cycles, lacking the pausing events seen at pol II genes^55^. Although pol III initiation complexes are extremely stable (also illustrated by the footprints at the TSS, Figs 1, 2 and 4), the high transcriptional rates are maintained by an efficient termination signal, which is essential for the rapid re-loading and recycling of pol III onto the template (i.e. the re-initiation)^56^,^57^. Therefore, regulation of Type II genes by the presence of a nucleosome at the TTS might rapidly affect transcriptional output by blocking the termination-dependent re-initiation. As previously mentioned, the RSC chromatin remodeler specifically controls access to the termination sequence through active remodeling of the +1 nucleosome at yeast tRNAs^17^,^23^,^30^, thereby fine-tuning the transcriptional output of the gene. In our study, the variable positioning of the +1 nucleosome across the termination site and/or internal promoter elements clearly depicts a similar model in transcriptional regulation of human tRNAs. However, we also show that regions upstream of the TSS appear to be important in the transcriptional regulation and expression of other pol III genes (Fig. 5 and Fig. S6). Additionally, the general lack of nucleosomes in pol III gene bodies, which are quite short, may allow high transcription rates, as nucleosomes are barriers to initiation, especially the +1 nucleosome, which can cause stalling and backtracking of the polymerase^38^,^58^. Furthermore, the weak positioning of the +1 nucleosome within, and between, cell populations at Type II promoters may be due to the dynamic expression of pol III transcripts through the cell cycle^35^,^59^,^60^. A previous study reported that the strong positioning of the −1 nucleosome at U6 was lost during mitosis^61^, which we do not observe in our data (Fig. 3). Hence, similar to studies at pol II genes^41^, future studies into the specific chromatin states and positioning patterns of pol III genes throughout the cell cycle would be of interest. Collectively, our data supports the concept that pol III genes are actively regulated by the chromatin environment, but also illustrate that further experiments in human cells are warranted to elucidate the cause and consequence of the differential positioning.

Previous work in yeast indicated that strong positioning and phasing of upstream nucleosomes was a distinctive feature of active pol III genes^9^,^30^,^62^. Likewise, we report strong positioning of upstream nucleosomes at active pol III genes in human cells (Fig. 1), which, however, is not unique to pol III genes^63^. The lack of heterogeneity in pol III NDR sizes is most likely the result of the strict promoter element usage (Type I–III promoters) at pol III genes that allows for little variability. However, despite this, the phasing of nucleosomes in both directions with a defined NDR anchor, which is much stronger and homogeneous than at pol II genes, makes this feature unique to pol III genes. Hence, active pol III genes punctuate the genome with these regions of anchored phasing. There have been multiple studies in yeast and human cells indicating that the strong NDR anchor and phasing characteristics around active tRNA genes could be involved in an insulator or barrier function^9^,^13^,^64^,^65^,^66^,^67^,^68^. As many pol III genes are found within enhancers or enhancer-like regions^20^,^26^,^27^,^28^,^52^,^69^, future experiments should aim to uncover if active pol III genes function as insulators within pol II enhancers and if this affects pol II regulated gene expression.

Collectively, our data provides an insight into the chromatin accessibility and nucleosome occupancy and positioning patterns at diverse pol III genes in human cells. Future studies into the remodelers and specific promoter elements involved at each pol III gene subset will help uncover how pol III transcription is fine-tuned and regulated, and how perturbations can be linked to states such as carcinogenesis.

## Methods

### Cell culture

K562 cells were cultured in Iscove’s Modified Dulbecco’s Medium, RKO and IMR90 in Eagle’s Minimum Essential Medium, HL60 in RPMI 1640 medium with Glutamax-1, T24 in McCoy’s 5a Medium, all supplemented with 10% FBS, 100 U/ml penicillin and 100 μg/ml streptomycin. Independent duplicate or triplicate experiments were performed for all cell lines, and cells were harvested during the exponential growth phase.

### Nucleosome footprinting (NOMe-seq) assay

Treatment of nuclei with M.CviPI was performed as previously described^29^. In brief, nuclei were isolated and subjected to M.CviPI, which methylates cytosines in GpC dinucleotides that are not protected by nucleosomes or tightly bound chromatin associated proteins, and DNA was isolated using phenol chloroform extraction. For salt treatment of nuclei for NOMe-seq, the protocol was modified with the addition of a 2 minute incubation at 4 °C with high salt buffer (10 mM Tris pH 7.4, 400 mM NaCl, 3 mM MgCl_2_, 0.1 mM EDTA, 250 mM sucrose) immediately after isolation of the nuclei prior to M.CviPI treatment.

Due to low GpC content of certain regions, we further modified this protocol to combine both M.CviPI and M.SssI enzymes in order to increase resolution of endogenously unmethylated sites. Briefly, for the combination treatment, freshly isolated nuclei were incubated with 300 units of M.CviPI, 5 ul 10X enzyme buffer (NEB), 1, 5 ul SAM (NEB), and 45 ul 1 M sucrose for 10 minutes at 37 °C. Next, 10 mM MgCl_2_, 1, 5 ul SAM and 50 units M.SssI (NEB) were added. The sample was incubated for an additional 10 minutes at 37 °C before the reaction was stopped (20 mM Tris-HCl, 600 mM NaCl, 10 mM EDTA, 1% SDS). The DNA was treated with proteinase K (NEB) overnight at 55 °C and DNA was purified using standard phenol chloroform extraction and then bisulfite converted (Zymo Research).

For loci-specific analysis, PCR was performed using primers that do not contain CpG or GpC sites, followed by gel purification (Qiagen), TA cloning (Invitrogen), plasmid amplification using Templiphi (Illustra) and Sanger sequencing. Pink bars denote regions of continuous inaccessibility of ≥146 bp, hence indicating the presence of a nucleosome. Regions of continuous inaccessibility >25 bp, but <146 bp around the TSS or TTS are indicated as footprints by orange bars. Prior to utilizing the modified protocol, the endogenous methylation status of the region was analyzed in each cell type to ensure an unmethylated state (Figs S3 and 4). Primer sequences are listed in Table S2.

### Library preparation

Genome wide NOMe-seq libraries were prepared using 2–3 μg of M.CviPI-treated DNA as previously described and sequenced on HiSeq 2500^29^,^70^.

### Genome wide data analysis

The library was sequenced on Illumina Hiseq 2500, with 75PE reads. 819106996 reads were sequenced, and 757776380 reads were mapped (93% mapping rate), and used to call for methylation. The bisulfite inconversion rate was 0.9%. NOMe-seq sequencing reads were aligned to the hg19 genome using BSMAP (Xi 2009). Methylation levels of CpG and GpC dinucleotides were determined using Bis-SNP and previously described parameters^63^,^71^. ChIP-seq data for pol III and pol II peaks were downloaded from ENCODE^52^ (http://genome.ucsc.edu/ENCODE/); all data used in this study is past the 9-months moratorium. Z-score for each ChIP-seq data was calculated as previously described^63^. TSS annotation was obtained from UCSC knownGenes track and a list of tRNAs was obtained from UCSC tRNA Genes track. All genome-wide data analyses were performed using previously described Bis-tools package^63^.

### RNA extraction and RT-qPCR

RNA was extracted using the miRNeasy Mini Kit (Qiagen) with on-column DNase digestion according to the manufacturer’s instructions. RNA was reverse transcribed using SuperScript III reverse transcriptase (Invitrogen) with Oligo-dT and random hexamers according to the manufacturer’s instructions.

RT-qPCR was performed using custom TaqMan probes (Applied Biosystems) for vtRNA and nc886 transcripts, GAPDH was quantified using SYBR green (Roche) on the LC480 machine (Roche). Primer sequences are listed in Table S3.

### Data access

NOMe-seq data generated in this study have been deposited to the NCBI Gene Expression Omnibus (GEO) under the accession number GSE64929.

## Additional Information

**How to cite this article**: Helbo, A. S. *et al*. Nucleosome Positioning and NDR Structure at RNA Polymerase III Promoters. *Sci. Rep.* **7**, 41947; doi: 10.1038/srep41947 (2017).

**Publisher's note:** Springer Nature remains neutral with regard to jurisdictional claims in published maps and institutional affiliations.

## Supplementary Material

Supplemental Information
